# Ending political abuse of psychiatry: where we are at and what needs to be done

**DOI:** 10.1192/pb.bp.114.049494

**Published:** 2016-02

**Authors:** Robert van Voren

**Affiliations:** 1 Human Rights in Mental Health-Federation Global Initiative on Psychiatry, The Netherlands

## Abstract

The number of reports of political activists falling victim to the political abuse of psychiatry is increasing. When the USSR first disintegrated, this practice virtually ceased to occur. What came in its place, however, was a disturbing collection of other forms of abuses, including human rights abuses, caused by a lack of resources, outdated treatment methods, a lack of understanding of individual human rights and a growing lack of tolerance in society. The number of cases of political abuse of psychiatry has increased since the 21st century began, particularly over the past few years in Russia, Belarus and Kazakhstan.

Over the past years, an increasing number of reports on the internment of political activists in former Soviet republics and particularly in Russia^1^ led to a resumed interest in the issue of the abuse of psychiatry for political purposes. Political abuse of psychiatry refers to the misuse of psychiatric diagnosis, treatment and detention for the purposes of obstructing the fundamental human rights of certain individuals and groups in a given society. The practice is common in, but not exclusive to, countries governed by totalitarian regimes. In these regimes abuses of the human rights of those politically opposed to the state are often hidden under the guise of psychiatric treatment. In democratic societies whistle-blowers on covertly illegal practices by major corporations have been subjected to the political misuse of psychiatry.^2^ Even though these abuses have been a frequent and ongoing practice throughout the 21 century in the People's Republic of China,^2^ that fact did not alert the world that this perversion of medical science has not come to an end. Rather, reports on individual cases of such abuses in former Soviet republics such as Belarus, Kazakhstan and Russia caught the attention and made people realise that 25 years after the conditional return of the Soviet psychiatric association to the World Psychiatric Association (WPA) the practice has still not been eradicated. Among the cases that have attracted wide public attention are those of the Pussy Riot band members (Russia), Mikhail Kosenko (Russia), one of the accused Bolotnaya Square protesters, who has been sentenced to mandatory treatment, and the psychiatric assessment of the Ukrainian pilot Nadezhda Savchenko, detained by the Russian government over the deaths of two Russian journalists in a mortar attack during the Ukraine conflict.

## Why abuse psychiatry politically?

The first question is why authorities resort to the internment of political or religious dissenters, or other types of ‘bothersome citizens’, in psychiatric hospitals. On the basis of 35 years of research and involvement in combating such practices, I have come to the conclusion that in most cases it is a combination of expedience and ideology.

Sending people to a psychiatric institution is particularly practical because hospitalisation has no end and thus, if need be, people can be locked away forever, or as long as they continue to have views that are considered politically or socially dangerous, or remain inconvenient to the authorities. One might think that such practices also exclude the need to have a lengthy pre-trial investigation and a bothersome court case, but often this is not true: dictatorial or totalitarian regimes tend to follow their own rules to the finest detail and document their repression meticulously, and thus in many cases the same legal procedures are followed as in the case when the person would be normally prosecuted and sentenced. Only in cases of short-term hospitalisations are these procedures sometimes bypassed, in particular when the internment is somewhere in the provinces out of the public eye and carried out to scare a person into submission or to settle an old dispute with a local authority.

At the same time, declaring a person mentally ill provides a perfect opportunity not to have to respond to their political or religious convictions, as they are the product of an ill mind and do not have to be taken seriously. In particular, when the views threaten or challenge the prevalent or only correct ideology (or religion), such a way out is especially welcome to the authorities, as one can maintain the claim that there is no opposition and one has a one hundred percent support of the population. As Soviet leader Nikita Khrushchev stated in 1959:

‘A crime is a deviation from the generally recognised standards of behaviour frequently caused by mental disorder. Can there be diseases, nervous disorders among certain people in Communist society? Evidently yes. If that is so, then there will also be offences that are characteristic for people with abnormal minds … To those who might start calling for opposition to Communism on this basis, we can say that … clearly the mental state of such people is not normal.’^3^

In the Soviet Union, the political abuse of psychiatry greatly benefitted from the fact that since 1950 only one view on psychiatry was permitted, which resulted in a virtually complete monopoly of the Moscow School of Psychiatry headed by Professor Andrei Snezhnevsky. Documentation shows that its leaders quite cynically allowed their profession to be used as a means of repression (see, for instance, a manuscript whose authors are kept anonymous for security reasons).^4^ However, the majority of Soviet psychiatrists were totally excluded from contact with international psychiatry and were truly convinced that people who opposed the Soviet regime were indeed suffering from mental illness and that their forced treatment was therefore correct and justified. In addition, the general tendency in society to comply with authoritarian orders out of fear of possible repercussions, such as the loss of jobs or income, played its role. Psychiatry was, in short, moulded into total subjugation to the needs of the existing political order.^5^

## Political abuse of psychiatry

### The former Soviet Union

From the moment the issue of political abuse of psychiatry attracted public attention in the late 1960s/early 1970s, it focused mainly on the Soviet Union. The systematic abuse of psychiatry in that country was a central issue during WPA debates in the period 1971-1989. It eventually led to the Soviet withdrawal from this international body in 1983 and an conditional return 6 years later (for an extensive discussion on the issues of Soviet psychiatric abuse and the reaction of world psychiatry, see Bloch & Reddaway^6,7^ and Van Voren^5^).

### Other countries

The Soviet Union was certainly not the only country where political abuse of psychiatry took place. Quite extensive documentation has been collected on similar abuses in other countries, notably some of the (socialist or communist) Eastern European countries. In particular, there have been extensive reports in the 1980s on systematic political abuse of psychiatry in Romania. Cases of abuse were also reported in Czechoslovakia, Hungary and Bulgaria, but these were isolated cases and there was no evidence that any systematic abuse took place. In the early 1990s an extensive research on the situation in Eastern Germany came to the same conclusion, although in this socialist country politics and psychiatry appeared to have been very closely intermingled.^8^

Outside of Eastern Europe and the former USSR, most reports concerned other socialist or collectivist societies, such as Cuba^9^ and the People's Republic of China.^2^ In China the abuse also involves people who are hospitalised for non-medical reasons because they are considered a burden owing to their constant complaints. Many victims are so-called ‘petitioners’, people who travel to Beijing from the provinces to issue complaints against local officials. Instead of being heard they are hospitalised and frightened with psychiatric ‘treatment’. Interestingly, a clear case of psychiatric abuse has been reported from The Netherlands, in the course of which the Ministry of Defence tried to silence a social worker by falsifying several of his psychiatric diagnoses and pretending his behaviour was the result of mental health problems.^10^ Still, one can conclude that the systematic use of psychiatry for political purposes is limited to countries that are characterised by a totalitarian monistic view and where dissent is seen as a threat that needs to be forced into the straightjacket of the existing political order.

## The post-Soviet period

When in 1991 the USSR imploded and all 15 Soviet republics gained or regained their independence, the dominance of Communism as the only permitted ideology ended. With it disappeared this monistic view on reality and thus also one of the main preconditions for the existence of a system of political abuse of psychiatry seemed to have vanished. And indeed, the practice of using psychiatry against political opponents virtually ceased to exist. Some cases surfaced, notably in 1996 in Turkmenistan and a few years later in Uzbekistan.^1^ What came in place, however, was a very disturbing collection of other forms of abuse, including human rights abuses owing to lack of resources, corruption, outdated methods of treatment, lack of understanding of individual human rights and a growing lack of tolerance in society. This includes ‘economic abuse’ (e.g. having relatives declared mentally ill in order to take control of their possessions) or criminals buying their way out to freedom by bribing psychiatrists to deliver false diagnoses, as well as general human rights violations in psychiatric institutions, such as adverse living conditions, abuse by staff, unlawful incarceration and inhumane treatment.

However, although on the outside the political climate in the former Soviet republics might have changed, in the minds of the citizens much of the psychological climate remained virtually unaltered. As a result, the effect of a monistic world view continues to dominate societies in most of the former Soviet republics, particularly in Central Asian republics, the Russian Federation and Belarus, and in some the communist ideology has been replaced by a nationalist or even neo-fascist world view that is as totalitarian as its predecessor.

Beginning with the 21st century, the number of individual cases of political abuse of psychiatry has increased, in particular over the past few years in Russia, Belarus and Kazakhstan.^1^ So far though, it does not appear to be a systematic repression of dissidents through the mental health system. In most cases, citizens fall victim to regional authorities in localised disputes or to private antagonists who have the means to bribe their way through the courts.

## How should psychiatry respond?

Interestingly, as a result of the debates concerning Soviet political abuse of psychiatry, the world psychiatric community focused much more extensively than before on issues of human rights, the rights of patients, issues of coercion and compulsory treatment. For instance, the WPA adopted an ethical code that condemns the use of psychiatry for non-medical purposes. This code was updated and expanded several times and mechanisms were installed to investigate complaints of violations of these regulations. The Hawaii Declaration of 1977 had been drawn up by the Ethical Sub-Committee of the Executive Committee of the WPA set up in 1973 in response to the increasing number of protests against the use of psychiatry for non-medical purposes. One of the principles embedded in the Declaration was that a psychiatrist must not participate in compulsory psychiatric treatment in the absence of psychiatric illness, and there were other clauses that could be seen as having a bearing on the political abuse of psychiatry. The Declaration was amended in Vienna in 1983 and in 1996 succeeded by the Madrid Declaration of 1996, which was further expanded in 1999. In addition, the WPA set up committees on ethics and on the review of abuse of psychiatry. In that sense, the debates surrounding Soviet psychiatric abuse had a very important corrective effect on world psychiatry, but that did not stop authorities – and psychiatrists – altogether from using psychiatry as a means of political repression.

At this moment, most of the political abuses reported are from communist or formerly communist states, notably the People's Republic of China and several former Soviet republics. However, on a positive note, international protests regarding cases of political abuse might have stopped the expansion of these practices and one of the most well-known victims, Mikhail Kosenko, was released from mental hospital already 6 months after his hospitalisation started, a clear sign that international attention works.^11^

In the 1970s and 1980s, the main drive of the opposition to Soviet psychiatric abuse was focused on expulsion of the Soviet society from the world psychiatric community, notably the WPA. In that case it worked, as loss of face played an important role in terminating the abuse. Importantly, rank and file psychiatrists did not suffer from this isolation, as they had no access to the world psychiatric community anyway. In a 21st-century society such total isolation is impossible, whatever measures authorities in countries such as China and Russia take to curb freedom of information, access to the internet and the use of social media.

Mental health professionals are now, at least in theory, able to have access to and participate in the world mental health community. Thus, the opposite approach to cutting off communication might now be effective: stimulating communication and access, providing training in issues of medical ethics and human rights, and translating key documents and manuals into local languages may make it impossible for the public to remain uninformed. In the case of the Russian Federation, a key element in the continued dominance of the Moscow School lies in the fact that 80–90% of the rank and file Russian psychiatrists do not know any second language. Therefore, when books, articles and documents are not available in Russian, it remains possible for the psychiatric leaders (many of whom hail from Soviet times) to pretend that the diagnosis used for Soviet dissidents – ‘sluggish schizophrenia’ – is quite accepted in the world and even part of ICD-10.^12^ Information is a weapon, and should be used maximally and extensively.
